# Learning from Wildfire Disaster Experience in California NICUs

**DOI:** 10.3390/children7100155

**Published:** 2020-10-01

**Authors:** Amy L. Ma, Ronald S. Cohen, Henry C. Lee

**Affiliations:** 1 Department of Pediatrics, Stanford University, Stanford, CA 94305, USA; rscohen@stanford.edu (R.S.C.); hclee@stanford.edu (H.C.L.); 2 California Perinatal Quality Care Collaborative, Stanford, CA 94305, USA

**Keywords:** NICU, perinatal care, California, wildfire, disaster preparedness, evacuation

## Abstract

Wildfires have been affecting California greatly, and vulnerable patients in neonatal intensive care units (NICUs) are not exempt. Our aim was to learn how personnel working in NICUs of California hospitals handled issues of neonatal transfer during wildfire disasters in recent years, with an ultimate goal to share lessons learned with healthcare teams on disaster preparedness. We identified California fires through newspaper articles and the CalFire.gov list. We determined which hospitals were affected and contacted members of the healthcare team through connections via the California Perinatal Quality Care Collaborative (CPQCC) database. We audio recorded interviews over phone or remote conferencing software or by written survey. We coded and analyzed transcripts and survey responses. While describing disaster preparedness, equipment (such as bassinets and backpacks), ambulance access/transport and documentation/charting were noted as important and essential. Teamwork, willingness to do other tasks that are not part of typical job descriptions, and unconventional strategies contribute to the success of keeping NICU babies safe when California wildfire strikes. Healthcare teams developed ingenious and surprising ways to evacuate NICU babies.

## 1. Introduction

“The 2018 wildfire season was the deadliest and most destructive wildfire season on record in California with a total of 8,527 fires” [1]. These wildfires led to various evacuations in healthcare settings including neonatal intensive care units (NICUs), and evacuation protocol reevaluation. Perinatal patients may be critically ill and highly dependent on medical staff and technology for care [2]. Babies cared for in NICUs are most likely to be impacted by acute evacuation from their units [3].

In addition to wildfires, it is inevitable that other types of disasters will also impact California healthcare systems in the future. The California Association of Neonatologists (CAN) resource, the “Neonatal Disaster Preparedness Toolkit,” addresses a plethora of forms of disaster preparedness from bioterrorism to an active shooter [2]. With the purpose of “provid(ing) guidance to NICU leadership in developing comprehensive disaster response plans that are in compliance with Joint Commission Standards and based on community, best-practice models” [2], the toolkit delves into the command center, six critical elements of disaster response [4] and the TRAIN™ tool (triage by resource allocation for in-patients).

The TRAIN™ tool is a way that NICU leaders categorize the infants under their care every day to prepare for evacuation if needed [5]. TRAIN™ tool assigns ambulance asset needs but not NICU level [2]. The assignments for transport can be car, BLS (basic life support), ALS (advanced life support), CCT (critical care transport), or Specialized with the categories being life support, mobility (car/car seat, stretcher, incubator, immobile), nutrition, and pharmacy [2]. The TRAIN™ tool provides a more subjective and efficient way to transport inpatients during a disaster evacuation [5]. We utilized the TRAIN™ tool because it has been studied and researched in its original form, as well as modified to be applicable to all neonatal and pediatric inpatients [5]. It is also a triage tool that meets the three needs of evacuation, surge capacity, and communication [5] and is endorsed by the CAN and the District IX AAP (American Academy of Pediatrics) section on Perinatal Pediatrics. Unlike the TRAIN™ tool, other available triage tools do not meet the three needs [5]. For this reason, our study exclusively used and focused on the TRAIN™ tool (Table 1).

Although guidelines have been published both in and out of California for various disasters, there is a gap in our knowledge in how these guidelines are implemented in actual disaster settings, particularly in the context of the recent wildfires in California. Additionally, we wanted to study the experiences and the practices of NICU personnel during evacuation or acceptance of babies. What are some of the barriers in healthcare delivery for NICUs, especially if needing evacuation, that we may have learned from recent California wildfire experiences? What have healthcare team members and leaders learned about how to improve patient safety during a crisis like a wildfire that is encroaching on the hospital’s door? What new ways have healthcare workers discovered and are enacting to effectively and efficiently evacuate NICUs? We answered these questions in an open-ended fashion as we wished to learn what health care team members were doing. These questions do not have absolute requirements based on certain guidelines.

In order to answer these questions, we researched NICUs that evacuated or accepted babies due to a California wildfire, contacted key members of the healthcare team (NICU medical directors, neonatologists, nurse managers/directors, neonatal clinical nurse specialists, NICU nurses, patient care manager, and NICU department managers), and conducted interviews from April 2019 to May 2020. The purpose of this study was to learn more about the issues faced by NICU healthcare providers during wildfire disasters, with the longer-term goal of improving NICU patient safety. We focused on NICUs that evacuated, as they made more of our sample pool of interviews.

## 2. Materials and Methods

We researched California fires using CALFire, newspaper articles, and internet search engines. Through newspaper articles and internet search engines, fifty-nine NICUs were investigated and a total of seven hospitals’ health care worker(s) were interviewed. The NICUs were spread out geographically and included parts of Northern and Southern California where the wildfires happened. Two interviewee’s NICUs are located in Southern California. Eight interviewee’s NICUs are located in Northern California, with six in the same city of Northern California. Of the six in the same city, hospital A had two interviewees, hospital B had one interviewee, and hospital C had two interviewees/three interviews, as one health care team member was interviewed twice due to two separate wildfires in different years affecting the same hospital.

We obtained contact information for the health care team members at these NICUs through contacts from the California Perinatal Quality Care Collaborative (CPQCC), referrals from previous participants that were interviewed, and other relevant sources. CPQCC is a California network of NICUs and HRIF (high risk infant follow-up) clinics, whose goal is to improve care for California’s mothers and most vulnerable infants [6]. We did not obtain contact information from HRIF clinic sources.

Once the informant was identified, we audio recorded approximately 60-min interviews over phone or remote conferencing software. We also gained additional information through online surveys. Of the ten interviews conducted from April 2019 to May 2020, three were obtained only through online survey. The online survey questions on Google Form were the same as the questions asked by phone or remote conference software and were reserved for participants with scheduling conflicts.

This qualitative study consisted of ten questions. By interviewing key members of the health care team, we were able to assess key components of patient safety in the hospital related to wildfire disaster response, specifically in regard to improving newborn care. A semi-structured interview process was used (Appendix A—interview guide). The interview questions contained four major sections: Participant’s Background, Institutional Perspective, Evacuation Experience, and Lessons and Insights. Please see Appendix A for the interview protocol.

Data were analyzed using qualitative research methods. The audio recordings were transcribed and read iteratively to find common themes in the interviews. We used a grounded theory approach based in “data systematically gathered and analyzed” but primarily focused on practical aspects that could be potentially generalizable to further work in the area of NICU preparedness for disaster experiences [7].

Before interviewing participants, interviewees filled out a consent and demographic form. All questions on the demographic form were optional. After the interviews, we emailed each participant a USD 25 gift card in appreciation for their time and effort. This study was approved by the Stanford University Institutional Review Board.

## 3. Results

Of the ten interviews, roles included NICU medical directors (*n* = 1), neonatologists (*n* = 2), nurse managers/directors (*n* = 2), neonatal clinical nurse specialists (*n* = 1), NICU nurses (*n* = 1), patient care managers (*n* = 1), and NICU department managers (*n* = 2). Overall, there were seven phone/remote conferencing software interviews and three online survey interviews. One participant was interviewed twice as their institution’s NICU was evacuated twice in different years.

While NICU level of care was not a focus of the questioning, the level of care came up in three of the interviewee’s responses. One was Level II, one Level III, and one Level IV. Each interviewee was interviewed on their own. However, as stated in the Materials and Methods section, two people were interviewed from the same location for two of the locations in Northern California.

### 3.1. NICU Redundant Systems

**Figure 1:**
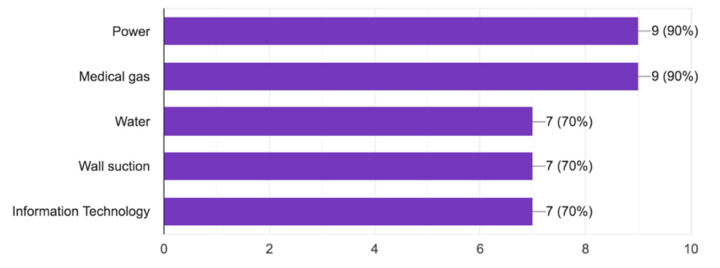
Neonatal intensive care units (NICU) redundant systems.

As seen in Figure 1, redundant systems were in place for power in 90%, medical gas in 90%, water in 70%, wall suction in 70%, and information technology in 70%.

### 3.2. Use of TRAIN™ Tool Nomenclature to Categorize Infants and Evacuation Preparedness

Table 2 shows the prevalence of TRAIN™ tool nomenclature in NICUs, hospitals, and regions to categorize infants. It also depicts evacuation preparedness including having necessary supplies and an established evacuation plan.

### 3.3. Please Describe Your Experience with [The Fire Incident] and the NICU Evacuation

When participants were asked about their NICU Evacuation Experience, 50% of participants declared that smoke and air quality were an issue. Two participants alleviated the bad air quality by using air scrubbers to pull the particulates out of the air.

In terms of transport, 40% used ambulances, with three participants using bassinets in the back of ambulances to transport babies. Other babies were triaged by bus or private car transport. One participant spoke of evacuating babies that could be feasibly transported in a car seat to be transferred in that way, in order to increase the capacity of the transport system for sicker babies.

For supplies, two participants spoke of the use of backpacks; one participant was putting together a small backpack per baby bedside including formula, wipes, diapers, and feeding tubes.

Communication to alleviate confusion was lacking in some NICUs. In one instance, babies showed up at the receiving hospital without a call from the evacuating NICU asking for permission or providing warning. Internet and phone call issues did not help. One participant had to text or text someone else who had access to the other person to communicate.

Furthermore, delegation of responsibilities was problematic. One participant said if they could have changed one thing, it would be to have a clear command structure in the unit.

Documentation of baby’s care was sometimes nonexistent by the receiving hospital. Compatibility of the electronic medical record (EMR) made a difference because it forced some medical professionals to have to chart on paper or wait for the nurse from the receiving hospital to chart for them. EMR incompatibility is an existing inefficiency even outside of a disaster context and is amplified even further when wildfire disaster strikes.

**Figure 2:**
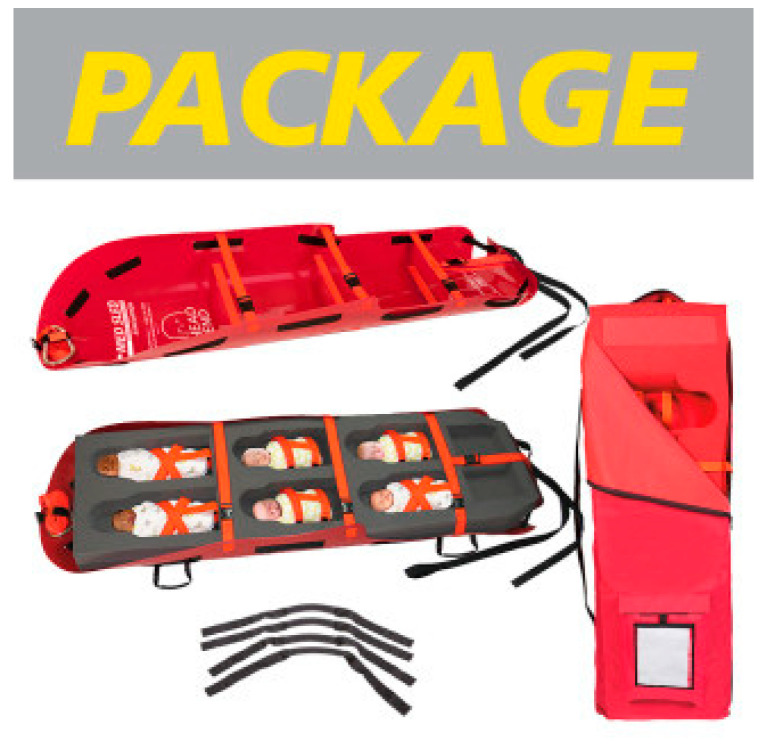
Med Sled^®^ Infant 6 insert.

**Figure 3:**
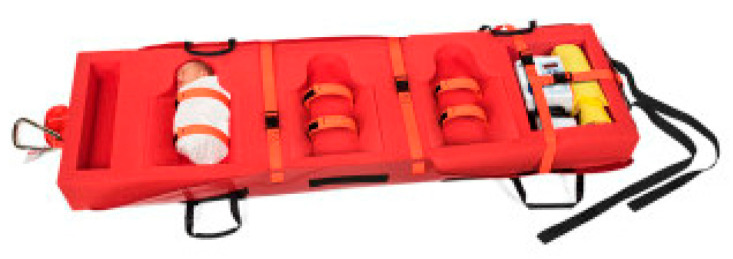
Med Sled^®^ infant insert that can hold up to three infants.

Forty percent of hospitals solved the problem of getting numerous babies out of the hospital at a time by using evacuation Med Sleds^®^. The Med Sled^®^ Infant 6 can fit up to six babies in the pockets (Figure 2) and another can fit three babies on a sled (Figure 3) and quickly get them out the door into an ambulance. The sleds lock in place, removing worry about the babies falling out. One hospital even noted how they could place the ventilator in the sled for a baby that needed it. This is one of other potential solutions to navigating babies going down the stairs, if the elevators are not working, an important component to evacuation.

Some participants took away getting as much supplies as they could if a disaster were to strike again and cause a NICU to evacuate; one hospital specified grabbing formula bottles and nipples. Two participants spoke of backpacks, with one putting together a daypack, a small backpack for each baby in case of emergency with formula, wipes, diapers, and feeding tubes and another revamping their backpacks.

Important to note in one hospital is that even though they tried ensuring people working in the NICU went through a streamlined vetting process, this did not happen for a number of people. This was an identified gap for future implementation work.

Infant identification was another concern by some of the hospitals. Ways to solve this problem that arose were making sure babies had ID bands on and stickers on their abdomens. A backup baby identification strategy may be important for local policies.

A theme from two informant interviews was limited storage space in the unit. One hospital did not keep the bassinets and incubators at hand, instead storing them in a couple of vacant patient rooms. Another hospital found it difficult to get equipment and supplies, including emergency food storage (formula), when the elevators are being used constantly and the hospital is chaotic.

Additionally, some participants explained the experience as traumatic or emotional with four participants relaying personal family issues including being evacuated or their house burning down as they were simultaneously helping NICU babies during the fire incident. Health care workers are resilient and selfless. Even though their own homes were burning down, they helped NICU babies stay safe and healthy.

### 3.4. Can You Describe Any Changes That Your NICU Implemented in Your Evacuation Procedures after the Experience with This Incident?

In terms of changes the participant’s NICUs implemented in their evacuation procedures after the experience with their corresponding fire incident, equipment was important to 50% of participants. Two participants cited bassinets for the perinatal patients, some with the ability to have oxygen attached to them. Another two participants spoke of aprons to carry babies, with one hospital realizing that they did not work well during their own evacuation.

### 3.5. Are There Other Aspects of Disaster Preparedness That You Think are Gaps in Your NICU? (Source: NICU Disaster Preparedness Survey—Gap Analysis)

When asked of gaps still existent in their NICU’s disaster preparedness, two respondents said it was staffing. There were minimal or skeleton staff during the wildfire, and they could not rely on people on call because those workers could not always get to the hospital. Furthermore, a common theme was the evacuation plan. One hospital noted that they had a pretty solid evacuation plan while another said that they are working on an evacuation plan and need two plans: one for horizontal for issues in NICU only (fire, structural issues, etc.) and one for vertical (out of building).

Two participants were confident in their disaster preparedness as the previous incident went smoothly and there were no gaps in protocols or procedures. Among the other 80%, one respondent explained how you identify gaps as you go and adapt. Big gaps included not using all resources to the fullest extent, getting patients out, getting equipment, not using the TRAIN™ tool for a live event or not having the TRAIN™ tool work, and making sure people were participating in drills and training for these disasters even though it is “not that likely for evacuation to happen again.” Another interesting insight was that the participant learned that they should have called the operation center instead of calling other potentially receiving NICUs themselves for better regional coordination and overlap prevention.

### 3.6. Can You Share Any Insights on How to Better Prepare for Future Disasters that Can Inform Neonatal Transport?

The insights that participants had on how to better prepare for future disasters that can inform neonatal transport include 30% declaring practicing is important, for example by doing live drills every year. One participant has not started doing drills yet. Two participants specified coordinating with their county/region with the county or statewide drills and spoke of having necessary equipment. Safety was something that one participant brought up, citing the fact that their use of open cribs in the ambulance could have been safer, so the babies do not have the chance of sliding around, even though their open cribs did not slide around in their fire experience.

A major insight brought up by one respondent was about being flexible in terms of what you are doing even though it does not meet your job description. Being open minded and thinking outside the box about how to transport babies, even putting them in car seats for example, is very important. Staying calm was another important insight by a participant who said that their neonatologist and nurses were very calm and acted fast.

### 3.7. Is There Anything Else That You Would Like to Share?

When asked for additional things to share, three spoke of community and a culture of team, with two specifying that even though the experience was personally traumatic with two of the team’s main people: their neonatologist and manager’s houses burning down, they still stepped up and showed up for their priorities. Staff did everything that needed to be done, some staying 48 h or longer. One participant is looking for the best models and is trying to teach that model to other areas, even implementing that model statewide as a possibility.

## 4. Discussion

With climate change, the incidence of wildfires that may lead to NICU evacuations are likely to increase. Therefore, it is important to be prepared and have effective plans in place to keep the most vulnerable population of babies, their caregivers, and medical staff as safe and healthy as possible [8].

Gaps in disaster preparedness exist and need to be identified, addressed and closed [9]. An example of a logistical gap is the inability to access temporary hospital privileges to “continue to take care of his or her patients in the receiving NICU” [10]. Congress and President George W. Bush saw gaps in disaster preparedness as a significant problem and established The National Commission on Children and Disasters in 2007 to try to close these gaps through a “cohesive national strategy… to protect children during disasters” [9].

These disasters include hurricanes. In August of 2005, Hurricane Katrina necessitated evacuation of NICUs as well. Dr. Barkemeyer explained what happened to the babies, hospital, city, trainees, and family while he was “on duty in the NICU of a flooded downtown New Orleans hospital” [11]. As a result of Katrina, babies were triaged and transported to other hospitals and a preterm baby girl was born four days after Katrina’s landfall and cared for using equipment on a portable generator [11]. Similar to our interviews focused on wildfires, communication methods following Hurricane Katrina was an issue [11].

Clearly, disaster preparedness also applies to events that are not wildfires, which is further shown in published guidelines and resources with hopes of keeping the NICU population safe [8]. The Illinois Department of Public Health released the “Neonatal Intensive Care Unit (NICU) Evacuation Guidelines,” planning in advance for transporting the NICU population and focuses on tornados, winter storms, flooding, and earthquakes (Illinois). The Illinois Guidelines suggest identifying infants in multiple ways, such as standard ID bands and direct patient marking, as well as performing horizontal evacuation before vertical evacuation [12]. The New York City Pediatric Disaster Coalition and New York City Department of Health and Mental Hygiene made a broad template available online titled the *Neonatal Intensive Care Unit Surge and Evacuation Plan* in 2018 [4]. However, descriptions of evacuation plans are adapted in the moment, including “using a smartphone to take pictures of patient arm bands” to facilitate tracking [13].

**Figure 4:**
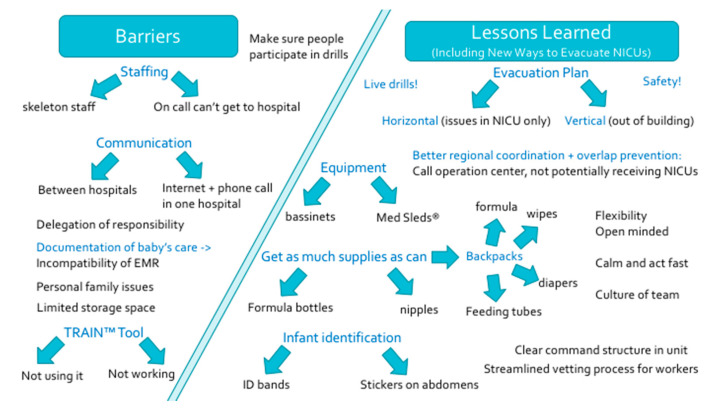
Barriers and lessons learned from wildfire disaster experience in California NICUs.

Common themes of our study are shown below in Figure 4. This figure answers the leading questions we posed in the introduction: “What are some of the barriers in healthcare delivery for NICUs, especially if needing evacuation, that we may have learned from recent California wildfire experiences? What have healthcare team members and leaders learned about how to improve patient safety during a crisis like a wildfire that is encroaching on the hospital’s door? What new ways have healthcare workers discovered and are enacting to effectively and efficiently evacuate NICUs?”

A limitation of our study is that we did not have knowledge of actual transfer of babies to which hospitals as this was a qualitative study, and we did not have patient data. There may also have been recall bias given the amount of time that had elapsed since the disasters that the interviewees discussed. Interviews were conducted from April 2019–May 2020. It is important to give health care members time to recover from crises and grieve, as these disasters can exert a heavy burden not only professionally but in personal lives. We aimed to have a balance in order to interview participants at a reasonable time in order to allow for an optimal recounting of the wildfire event while not causing undue stress.

Disaster resources for center internal evaluation of their own processes include the California Association of Neonatologists’ Neonatal Disaster Preparedness Toolkit and Stanford’s Disaster Planning Toolkit, both linked below.

1. https://www.cpqcc.org/sites/default/files/pdf/toolkit/CAN_Neonatal%20Disaster%20Preparedness%20Toolkit_02.2015.pdf
2. https://obgyn.stanford.edu/divisions/mfm/disaster-planning.html

## 5. Conclusions

Overall, the recent history of neonatal intensive care unit (NICU) evacuations due to wildfires in Northern and Southern California provided an opportunity to learn from past disaster experience and to be better prepared for the future. NICU teams have solved some issues faced during wildfire disaster, improving future disaster response. However, there is much more improvement that needs to be done. We hope our findings aid and inspire NICUs to reevaluate and possibly change their disaster preparedness protocol for evacuating due to a California wildfire. Health care team members are heroes. We need the system of disaster preparedness to improve, so our selfless medical community and vulnerable NICU patients have hope, resources, and practices to stay safe and healthy.

## Figures and Tables

**Table 1:** Triage by resource allocation for inpatients (TRAIN) tool. (Adapted from Dr. Lin’s *Triage by Resource Allocation for Inpatients: A Novel Disaster Triage Tool for Hospitalized Pediatric Patients*, 2018) [5].

Transport	Blue/Car	Green/BLS	Yellow/ALS	Orange/CCT	Red/Specialized
**Life Support**	Stable	Stable +	Minimal	Moderate	Maximal
**Mobility**	Car/Car seat	Wheelchair or Stretcher	Wheelchair or Stretcher	Transport rig	Incubatoror Immobile
**Nutrition**	All PO	Intermittent Enteral	Continuous Enteral or Partial Parenteral	TPN Dependent	
**Pharmacy**	PO Meds	IV Intermit meds	IV Fluids	IV Drip ×1	IV Drip ≥2

PO: Per os (taken orally); TPN: Total parenteral nutrition; IV: Intravenous medication.

**Table 2:** Use of TRAIN™ tool nomenclature to categorize infants and evacuation preparedness.

	Yes (%)
The California Association of Neonatologists and the District IX AAP section on Perinatal Pediatrics have endorsed TRAIN™ tool (triage by resource allocation for in-patients). Did your NICU use TRAIN™ tool nomenclature to categorize the infants under their care?	60%
Did your hospital use TRAIN™ tool nomenclature to categorize the infants under their care?	30%
Did your region use TRAIN™ tool nomenclature to categorize the infants under their care?	30%
Did your NICU have the necessary supplies for safe evacuation (including equipment stockpile such as emergency transport devices, backpacks, portable pulse oximeters, etc.)?	70%
Did your NICU have an established evacuation plan (including knowledge of how to stage the two types of evacuation—horizontal and vertical, determining priorities, established clear roles and responsibilities, rehearsal of these roles and ensuring quick and easy access to emergency equipment, supplies and documentation forms, emergency medication administration, and transport)?	80%

AAP: American Academy of Pediatrics; NICU: Neonatal Intensive Care Unit.

## Appendix A: Interview Protocol

### Participant’s Background

1. Please describe your role.
2. How long have you been in your present position?
3. How long have you been at this institution?
4. Briefly describe your role as it relates to the NICU evacuation that you will be describing?

### Institutional Perspective

5. Please describe the procedures that were in place in your NICU prior to the event:

Probes (Refer to NICU Disaster Preparedness Survey—Evacuation Planning):
  - Did your NICU have redundant systems for:
    Power?
    Medical gas?
    Water?
    Wall suction?
    Information Technology?
  - The California Association of Neonatologists and the District IX AAP section on Perinatal Pediatrics have endorsed TRAIN™ tool (triage by resource allocation for in-patients). Did your **NICU** use TRAIN™ tool nomenclature to categorize the infants under their care? No (1), Yes (2), Don’t Know (3).
  - Did your hospital use TRAIN™ tool nomenclature to categorize the infants under their care? No (1), Yes (2), Don’t Know (3)
  - Did your region use TRAIN™ tool nomenclature to categorize the infants under their care? No (1), Yes (2), Don’t Know (3)
  - Did your NICU have the necessary supplies for safe evacuation (including equipment stockpile such as emergency transport devices, backpacks, portable pulse oximeters, etc.)? No (1), Yes (2), Don’t Know (3)
  - Did your NICU have an established evacuation plan (including knowledge of how to stage the two types of evacuation—horizontal and vertical, determining priorities, established clear roles and responsibilities, rehearsal of these roles and ensuring quick and easy access to emergency equipment, supplies and documentation forms, emergency medication administration, and transport)? No (1), Yes (2), Don’t Know (3)

### Evacuation Experience

6. Please describe your experience with [the Fire Incident] and the NICU Evacuation.

### Lessons and Insights

7. Can you describe any changes that your NICU implemented in your evacuation procedures after the experience with this incident?
8. Are there other aspects of disaster preparedness that you think are gaps in your NICU? (Source: NICU Disaster Preparedness Survey—Gap Analysis)
9. Can you share any insights on how to better prepare for future disasters that can inform neonatal transport?
10. Is there anything else that you would like to share?
